# Association between kidney stones and urological cancers: results from the NHANES 2007–2020 and Mendelian randomization study

**DOI:** 10.1007/s12672-025-02415-4

**Published:** 2025-04-24

**Authors:** Jinghua Zhong, Jiahao Cheng, Zhijian Zhao, Houmeng Yang, Yongda Liu, Xiaolu Duan, Guohua Zeng

**Affiliations:** 1 Department of Urology, The First Affiliated Hospital, Guangzhou Medical University, Guangzhou, 510230 Guangdong Province China; 2 Guangdong Provincial Key Laboratory of Urological Diseases, Guangzhou Medical University, Guangzhou, 510230 Guangdong Province China; 3 Guangdong Engineering Research Center of Urinary Minimally Invasive Surgery Robot and Intelligent Equipment, Guangzhou Medical University, Guangzhou, 510230 Guangdong Province China; 4 Guangzhou Institute of Urology, Guangzhou Medical University, Guangzhou, 510230 Guangdong Province China; 5 Department of Clinical Medicine, Nanshan School, Guangzhou Medical University, Guangzhou, 511436 Guangdong Province China; 6 Department of Urology, Hwa Mei Hospital, University of Chinese Academy of Sciences (Ningbo No.2 Hospital), Ningbo, 315010 Zhejiang China

**Keywords:** Kidney stones, Renal cell carcinoma, Bladder cancer, Prostate cancer, NHANES, Mendelian randomization

## Abstract

**Background:**

Kidney stones is a common urological disease with a rising incidence in global. The association between kidney stones and urological cancers remains controversial. This study utilized the data from the 2007–2020 National Health and Nutrition Examination Survey (NHANES) and Mendelian randomization (MR) analysis to evaluate the association and potential causal relationship between kidney stones and renal cell carcinoma, bladder cancer, and prostate cancer.

**Methods:**

Multivariate logistic regression was used to examine the association between kidney stones history and urological cancers, followed by stratified analyses. Subsequently, causal relationships were explored via the inverse variance weighted (IVW), weighted median, and MR-Egger methods. Sensitivity analyses were performed to ensure the robustness of the findings.

**Results:**

Data from 13,013 individuals (5,138 males) were analyzed. Kidney stones was significantly associated with an increased risk of renal cell carcinoma (OR = 1.92, 95% CI 1.90–1.95, P < 0.001), bladder cancer (OR = 2.749, 95% CI 2.71–2.78, P < 0.001), and prostate cancer (OR = 2.03, 95% CI 2.02–2.04, P < 0.001). However, MR analysis did not provide evidence for a genetic causal relationship between kidney stones and these cancers. Sensitivity analyses confirmed the stability and reliability of the MR results.

**Conclusion:**

Kidney stones increased the risk of renal cell carcinoma, bladder cancer, and prostate cancer in the US population. MR analysis did not establish a genetic causal relationship between kidney stones and renal cell carcinoma, bladder cancer, and prostate cancer in the European population.

**Supplementary Information:**

The online version contains supplementary material available at 10.1007/s12672-025-02415-4.

## Introduction

Kidney stones is a common urological condition with a rising incidence in global and a high recurrence rate of 50% within 5 years following the initial treatment [1–3]. In the United States alone, medical expenditures related to kidney stones exceed 10 billion dollars annually, placing a significant economic burden on the healthcare system [4, 5]. Epidemiological and clinical studies also indicated that kidney stones may contribute to the development of hypertension, chronic kidney disease, and end-stage renal disease, highlighting it as a risk factor for multiple diseases [6–8].

Previous studies have reported inconsistent findings regarding the association between kidney stones and urological cancers. A Dutch cohort study of 120,000 individuals observed a significantly increased risk of renal cell carcinoma (OR = 1.39, 95% CI 1.05–1.84) and upper tract urothelial carcinoma (UTUC) (OR = 1.66, 95% CI 1.03–2.68) among patients with a history of kidney stones [9]. In contrast, a meta-analysis of seven studies revealed that this same conclusion was limited to male patients [10]. Additionally, three other studies beyond the aforementioned meta-analysis supported a correlation between kidney stones and an elevated risk of ureteral cancer and renal pelvis cancer [11–13]. Swedish researchers argued that kidney or urinary stones only elevate the risk of urothelial carcinoma but not renal cell carcinoma [14]. However, a case–control study from the United States found no association between kidney or bladder stones and bladder cancer [15]. Interestingly, a study based on a Taiwanese population database revealed that prostate cancer was associated with kidney stones (OR = 1.71; 95% CI 1.42–2.05), bladder stones (OR = 2.06; 95% CI 1.32–3.23), and multiple stones (OR = 1.73; 95% CI 1.47–2.02) [16].

Observational studies have suggested a potential association between kidney stones and urological cancers, but this relationship remains uncertain due to limitations in sample size and the influence of confounding factors. National Health and Nutrition Examination Survey (NHANES), a biennial survey of the American population, offers a large and representative data set collected through multi-stage probability sampling, interviews, questionnaires, physical examinations, and laboratory data [17]. Mendelian randomization (MR) analysis is a methodological tool that uses genetic variants as instrumental variables to investigate causal relationships between exposure factors and disease phenotype [18]. Given that genetic alleles are randomly allocated during meiosis and not influenced by environmental factors, MR analysis is superior to observational studies in controlling for confounding factors. Considering the substantial familial heritability of both kidney stones and urological cancers, MR analysis may serve as a valuable complementary approach [19, 20].

To investigate the association between kidney stones and urological cancers, we initially analyzed data from the NHANES, adjusting for potential confounders. Subsequently, bidirectional MR analysis was performed to further assess the causal relationship between kidney stones and renal cell carcinoma, bladder cancer, and prostate cancer.

## Methods

### Study population in the NHANES database

**Fig. 1:**
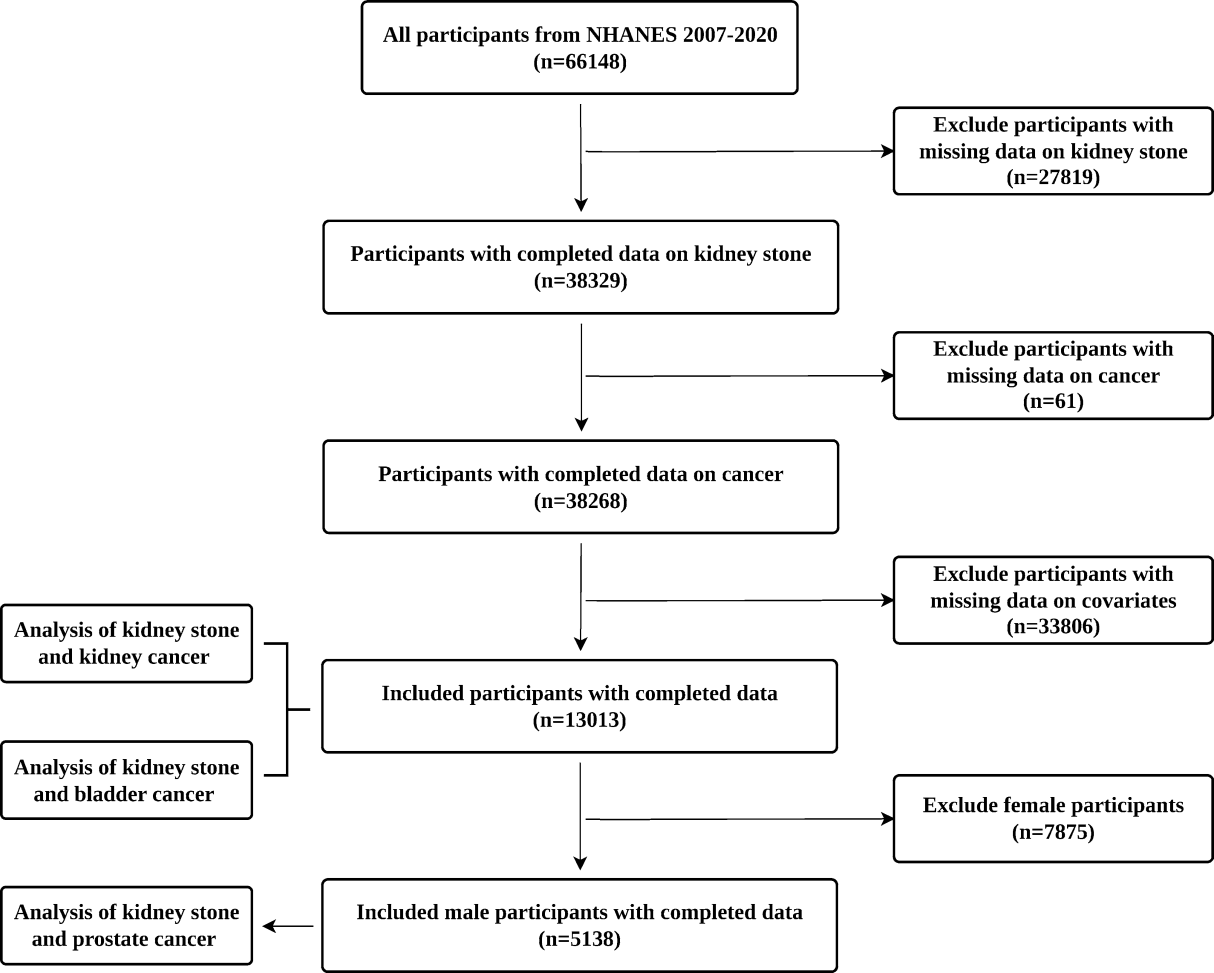
Flowchart of participant selection in the NHANES 2007–2020 study. No additional caption

This investigation analyzed the data from six cycles of the NHANES database, spanning the years 2007 to 2020 (2007-2008, 2009-2010, 2011-2012, 2013-2014, 2015-2016, 2017–2020). Adult participants (≥ 20 years) with complete data on kidney stones history and cancer history were included (n = 38,268). After excluding individuals with missing demographic information and covariates (n = 25,255), a total of 13,013 participants were retained for the analysis of the association between kidney stones and the incidence of kidney or bladder cancer. Furthermore, after excluding female participants, 5138 male participants were included to specifically examine the association between kidney stones and prostate cancer (Fig. 1).

### Collection of history of kidney stones and urological cancers in NHANES

The history of kidney stones was determined using the kidney condition questionnaire, which included the query, “Have you ever had kidney stones?”. Participants responding affirmatively were classified as having a history of kidney stones. Urological cancers history was obtained through the medical condition questionnaire, which included the questions, “Have you ever been diagnosed with cancer or malignancy?”. Participants responding affirmatively were subsequently queried, “What type of cancer?”.

### Covariate information in NHANES

To enhance the precision of our findings, we included the following covariates for model adjustment: age, gender, race/ethnicity (categorized as Mexican American, Other Hispanic, Non-Hispanic Black, Non-Hispanic White and Other Race), education level (categorized as high school graduate or below, some college, and college graduate or above), family income, body mass index (BMI), smoking history (more than 100 cigarettes in a lifetime), alcohol consumption history (at least 12 alcohol drinks in lifetime), and histories of diabetes, hypertension, and coronary heart disease. Family income was measured using poverty income ratio (PIR) and divided into three levels according to the Supplemental Nutrition Assistance Program cited in the 1999–2010 NHANES Analysis Guidelines [21]: 0–1.30 (the lowest income), 1.31–3.50 (the middle income), and 3.51–5.00 (the highest income). Chronic diseases such as diabetes, hypertension, and coronary heart disease were identified based on self-reported history.

### Statistical analysis for NHANES

Considering the complex multistage stratified sampling of NHANES, all statistical analyses were weighted according to NHANES guidelines [21–23]. For the overall characteristics of the study population, continuous variables were presented as mean ± standard deviation, while categorical variables were described as percentages. Statistical comparisons between groups were performed using weighted t-tests for continuous variables and weighted chi-square tests for categorical variables.

Subsequently, weighted multivariate logistic regression analysis were performed to examine the relationship between kidney stones and renal cell carcinoma, bladder cancer, and prostate cancer, with stepwise adjustments for potential confounders. Model 1 was unadjusted. Model 2 was adjusted for age, gender and race/ethnicity. Model 3 was fully adjusted for age, gender, race/ethnicity, education level, PIR, BMI, diabetes, hypertension, coronary heart disease, smoking behavior, and alcohol consumption.

Additionally, we performed subgroup analyses stratified by gender, age, race, education level, and PIR to investigate the association between kidney stones and the three types of urological cancer in different populations.

Data from the NHANES database were analyzed using IBM SPSS 25.0 software. Statistical significance was defined as a two-sided P < 0.05.

### Screening of genetic instrument for kidney stones in MR

**Fig. 2:**
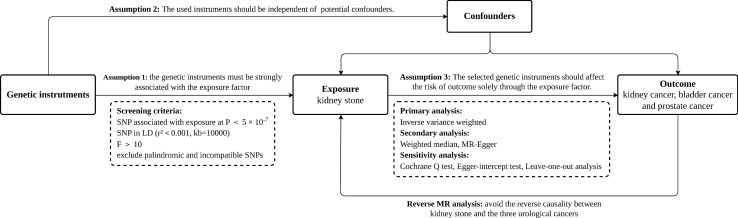
Framework for Mendelian randomization analysis. The genetic instruments are chosen based on three assumptions: (1) they must be strongly associated with the exposure (kidney stones), (2) they should be independent of potential confounders, and (3) they should affect the risk of outcomes (renal cell carcinoma, bladder cancer, and prostate cancer) only through the exposure. Primary and secondary MR analysis and sensitivity analyses are applied to ensure validity

The selection of instrument variables (IVs) for MR analysis based on the following three principles: (1) the genetic IVs must be strongly associated with the exposure factor; (2) the IVs are independent of other confounding factors; and (3) the IVs affect the outcome solely through the exposure factor [24]. The framework of the MR study design is depicted in Fig. 2.

The Genome-wide association study (GWAS) data for kidney stones were sourced from a study published by Handan Melike Dönertaş in 2021, comprising 3725 cases and 480,873 controls, including a total of 9,587,836 single nucleotide polymorphisms (SNPs) [25]. Given the limited number of significant SNPs, a significance threshold of P < 5 × 10^–6^ was established. Subsequently, SNPs in linkage disequilibrium were excluded (r^2^ < 0.01 within a 10,000 kb window). The F-statistic of each SNP was calculated to assess its association with the exposure, retaining SNPs with F-statistic > 10 which were considered as strong SNPs [26, 27]. Finally, harmonization was performed to exclude palindromic and incompatible SNPs. The detailed information of SNPs used for kidney stones is shown in Table 1.

**Table 1:** Characteristics of the genetic variants that were used as the instrumental variables for kidney stones

SNP	Beta	pval	SE	Effect allele	Other allele	EAF	F	Gene
rs10020631	0.00101743	1.10E-06	0.000209419	A	G	0.242862	23.60343025	TMPRSS11E,UGT2B17
rs10051765	0.00131957	2.60E-12	0.000188665	C	T	0.337459	48.9193181	F12,LMAN2,PFN3,RGS14,SLC34A1
rs115686032	0.00390442	2.20E-06	0.000826132	T	C	0.012372	22.33635871	NA
rs116245586	0.00305153	6.30E-10	0.000493478	G	A	0.034778	38.23824078	ALPL,NBPF3
rs11712086	− 0.00131956	3.50E-06	0.000285624	C	T	0.112847	21.34357175	CBLB
rs117181902	0.00271799	3.70E-06	0.000588112	A	G	0.025256	21.35865175	NA
rs117873120	0.00414991	3.20E-06	0.000892805	T	C	0.011524	21.60540125	GRIK4,TBCEL
rs13373881	0.0014212	1.10E-06	0.000292228	C	T	0.104489	23.65184116	LINC00853,PDZK1IP1,STIL,TAL1
rs1360768	0.000884079	7.60E-07	0.000178767	A	C	0.485455	24.45713906	NA
rs147254698	0.00425663	1.80E-06	0.000889986	A	C	0.011993	22.87513473	PRKD1
rs187292007	0.0044741	9.40E-07	0.000910859	G	A	0.011621	24.12721939	DNASE1,NLRC3,SLX4,TRAP1
rs1950845	− 0.0012663	4.20E-06	0.00027515	A	C	0.124597	21.18031365	NA
rs2776288	− 0.0012391	1.90E-11	0.000184703	G	A	0.382077	45.00523385	CHAF1B,CLDN14
rs2924814	0.000870044	1.20E-06	0.000178939	C	G	0.469579	23.6412605	DGKD,USP40
rs34554648	0.00215146	3.00E-06	0.000460977	G	A	0.039023	21.7824259	NA
rs416699	0.000936344	4.20E-06	0.000203434	T	C	0.732346	21.18468421	HSD17B3,SLC35D2
rs4388470	0.001951	1.80E-06	0.000408387	T	C	0.050062	22.82280021	NA
rs4924445	0.000853947	3.90E-06	0.000184976	A	G	0.392979	21.31224864	ANKRD63,C15orf56,PAK6,PLCB2
rs6127099	− 0.00129216	2.20E-10	0.000203439	T	A	0.279954	40.34245922	BCAS1,CYP24A1,MIR4756
rs62065454	− 0.000872144	1.20E-06	0.000179558	G	A	0.504145	23.5920176	LRRC37A4P,MIR4315-1,MIR4315-2,PLEKHM1
rs664777	− 0.0015828	1.50E-07	0.000300996	C	T	0.898372	27.65214607	ASAP3,C1orf213,HNRNPR,TCEA3,ZNF436
rs6794839	− 0.000964322	1.50E-06	0.00020042	G	A	0.274607	23.15049288	RARB
rs71606723	0.00109269	3.70E-07	0.000214953	T	A	0.221169	25.84075052	UGT8
rs73182697	0.00353313	4.90E-06	0.000772734	A	C	0.01436	20.90534791	LINC00547,POSTN,TRPC4
rs73187207	0.00108993	6.20E-07	0.000218703	T	G	0.211206	24.83624526	DGKH
rs760900	0.000869414	1.40E-06	0.000180056	G	A	0.546214	23.31504566	TFAP2B
rs7740107	0.00101406	6.20E-07	0.00020335	A	T	0.740134	24.86778812	L3MBTL3
rs77826930	0.0036167	1.90E-06	0.00075959	C	G	0.01392	22.67068814	TMEM132B
rs79670018	0.00110151	3.30E-07	0.000215813	T	C	0.249595	26.05072968	ZBTB43
rs8034840	− 0.000982968	2.20E-06	0.000207989	A	G	0.244813	22.33552852	NDUFAF1,NUSAP1,RTF1

*EAF* Effect Allele Frequency, *F* F statistic value, *NA* not available, *pval* p value, *SE* standard error, *SNP* single nucleotide polymorphism

### Outcome data sources in MR

The GWAS summary data for renal cell carcinoma were obtained from a GWAS meta-analysis, comprising 25,890 cases and 743,585 controls [28]. For bladder cancer, the data were sourced from Jiang's study, including 2264 cases and 454,084 controls [29]. From the PRACTICAL consortium, we obtained the GWAS data for prostate cancer, encompassing a total of 79,148 cases and 61,106 controls [30]. Table 2 summarizes all GWAS data and their sources included in this study.

**Table 2:** Sources of the GWASs included in the Mendelian randomization analysis

Trait	Cases	Participants	Web source
Kidney stones	3,725	480,873	https://gwas.mrcieu.ac.uk/datasets/ebi-a-GCST90038631/
Renal cell carcinoma	25,890	743,585	https://www.ebi.ac.uk/gwas/studies/GCST90320057
Bladder cancer	2,264	454,084	https://www.ebi.ac.uk/gwas/studies/GCST90041857
Prostate cancer	79,148	61,106	http://practical.icr.ac.uk/blog/

### Statistical analysis for MR

TwoSampleMR R package was used for MR analysis [31]. The genetic causal relationships between kidney stones and renal cell carcinoma, bladder cancer, and prostate cancer were primarily evaluated using IVW, weighted median, and MR-Egger. Sensitivity analyses were performed using the following methodologies. The Cochrane Q test based on IVW and MR-Egger method was utilized to assess potential heterogeneity among SNPs. The Egger-intercept test was conducted to detect directional pleiotropy. The MR-Pleiotropy RESidual Sum and Outlier (MR-PRESSO) method was applied to identify potential outliers and horizontal pleiotropy [32]. Moreover, the leave-one-out (LOO) analysis was performed to avoid the potential influence of any single SNP on the overall results. Finally, reverse MR analysis was conducted to exclude potential reverse causality.

All statistical analysis and data visualization were performed in R software (version 4.2.0). Two-sided P value < 0.05 was considered statistically significant.

## Results

### General characteristics of NHANES

Table 3 presents the baseline characteristics of the study population stratified by cancer type. Weighted chi-square analysis revealed significant associations between kidney stones and renal cell carcinoma, bladder cancer, and prostate cancer. Compared to the control group, males were significantly overrepresented in all cancer groups, and non-Hispanic Whites constituted the largest proportion across all racial groups. Regardless of urological cancer type, individuals with a history of smoking, alcohol consumption, diabetes, hypertension, or coronary artery disease were significantly more prevalent in the cancer groups. Tables 4 and 5 further stratify the study population base on the presense or absence of a history of kidney stones, and present the baseline characteristics of each group.

**Table 3:** Basic characteristics categorized by cancers

Characteristics	Renal cell carcinoma			Bladder cancer			Prostate cancer		
	Renal cell carcinoma (n = 31)	Non-renal cell carcinoma (n = 12,982)	P	Bladder cancer (n = 27)	Non-bladder cancer (n = 12,986)	P	Prostate cancer (n = 199)	Non-prostate cancer (n = 4939)	P
Kidney stones (%)									
Yes	9 (29.0%)	1272 (9.8%)	< 0.001	8 (29.6%)	1273 (9.8%)	< 0.001	40 (20.1%)	558 (11.3%)	< 0.001
No	22 (71.0%)	11,710(90.2%)		19 (70.4%)	11,713 (90.2%)		159 (79.9%)	4381 (88.7%)	
Age, mean ± SD, year	68.06 ± 12.51	51.65 ± 17.75	< 0.001	75.4 ± 5.26	49.15 ± 17.45	< 0.001	72.47 ± 7.25	50.46 ± 17.73	< 0.001
Gender									
Male	21 (67.7%)	5117 (39.4%)	< 0.001	19 (70.4%)	511,939.4%)	< 0.001	199 (3.9%)	4939 (96.1%)	–
Female	10 (32.3%)	7865 (60.6%)		8 (29.6%)	7875 (60.5%)		–	–	
Body mass index, mean ± SD, kg/m2	31.60 ± 5.81	29.97 ± 7.57	< 0.001	28.60 ± 6.29	29.91 ± 7.40	< 0.001	28.95 ± 5.70	29.19 ± 6.55	< 0.001
Race/ethnicity (%)									
Mexican American	7 (22.6%)	1771 (13.6%)	< 0.001	–	1778 (13.7%)	< 0.001	6 (3.0%)	587 (11.9%)	< 0.001
Other Hispanic	2 (6.5%)	1378 (10.6%)		1(3.7%)	1379 (10.6%)		14 (7.0%)	471 (9.5%)	
Non-Hispanic white	15 (48.4%)	4594 (35.4%)		21(77.8%)	4588 (35.3%)		103 (51.8%)	1820 (36.8%)	
Non-Hispanic black	5 (16.1%)	3232 (24.9%)		3(11.1%)	3234 (24.9%)		62 (31.2%)	1219 (24.7%)	
Other race	2 (6.5%)	2007 (15.5%)		2(7.4%)	2007 (15.5%)		14 (7.0%)	842 (17.0%)	
Level of education (%)									
High school or below	11 (35.5%)	6058 (46.7%)	< 0.001	10 (37.0%)	6059 (46.7%)	< 0.001	82 (41.2%)	2310 (46.8%)	< 0.001
Some College	14 (45.2%)	3970 (30.6%)		12 (44.4%)	3972 (30.6%)		60 (30.2%)	1441 (29.2%)	
College or above	6 (19.4%)	2954 (22.8%)		5 (18.5%)	2955 (22.8%)		57 (28.6%)	1188 (24.1%)	
Poor income ratio (%)									
< 1.3	5 (16.1%)	4175 (32.2%)	< 0.001	6 (22.2%)	4174 (32.1%)	< 0.001	34 (17.1%)	1409 (28.5%)	< 0.001
1.3–3.49	17 (54.8%)	5129 (39.5%)		15 (55.6%)	5131 (39.5%)		92 (46.2%)	1990 (40.3%)	
≥ 3.5	9 (29.0%)	3678 (28.3%)		6 (22.2%)	3681 (28.3%)		73 (36.7%)	1540 (31.2%)	
Smoking behavior (%)									
Smoker	16 (51.6%)	4322 (33.3%)	< 0.001	15 (55.6%)	4323 (33.3%)	< 0.001	96 (48.2%)	2169 (43.9%)	< 0.001
Non-smoker	15 (48.4%)	8660 (66.7%)		12 (44.4%)	8663 (66.7%)		103 (51.8%)	2770 (56.1%)	
Alcohol consumption (%)									
Drinker	27 (87.1%)	9172 (70.7%)	< 0.001	19 (70.4%)	9180 (70.7%)	< 0.001	162 (81.4%)	3885 (78.7%)	< 0.001
Non-drinker	4 (12.9%)	3810 (29.3%)		8 (29.6%)	3814 (29.3%)		37 (18.6%)	1054 (21.3%)	
Hypertension (%)									
Yes	23 (74.2%)	5131 (39.5%)	< 0.001	21 (77.8%)	5133 (39.5%)	< 0.001	132 (66.3%)	1824 (36.9%)	< 0.001
No	8 (25.8%)	7851 (60.5%)		6 (22.2%)	7853 (60.5%)		67 (33.7%)	3115 (63.1%)	
Diabetes (%)									
Yes	15 (48.4%)	2066 (15.9%)	< 0.001	9 (33.3%)	2072 (16.0%)	< 0.001	59 (29.6%)	811 (16.4%)	< 0.001
No	16 (51.6%)	10,916 (84.1%)		18 (66.7%)	10,914 (84.0%)		140 (70.4%)	4128 (83.6%)	
Coronary heart disease (%)									
Yes	5 (16.1%)	560 (4.3%)	< 0.001	3 (11.1%)	562 (4.3%)	< 0.001	30 (15.1%)	304 (6.2%)	< 0.001
No	26 (83.9%)	12,422 (95.7%)		24 (88.9%)	12,424 (95.7%)		169 (84.9%)	4635 (93.8%)	

Mean and standard deviation are used for continuous variables (e.g., age and body mass index), while proportions are used for categorical variables (e.g., gender and race/ethnicity). Chi-square test used to compare groups. p < 0.05 indicates significant difference

**Table 4:** Basic characteristics categorized by kidney stones (renal cell carcinoma and bladder cancer)

Characteristic	Kidney stones (n = 1281)	Non-kidney stones (n = 11,732)	P
Renal cell carcinoma (%)			
Yes	9 (0.7%)	22 (0.2%)	< 0.001
No	1272 (99.3%)	11,710 (99.8)	
Bladder cancer (%)			
Yes	8 (0.6%)	19 (0.2%)	< 0.001
No	1273 (99.4%)	11,713 (99.8%)	
Gender			
Male	598 (46.7%)	4540 (38.7%)	< 0.001
Female	683 (55.3%)	7192 (61.3%)	
Age, mean ± SD, year	54.29 ± 15.43	48.6 ± 17.59	< 0.001
Body mass index, mean ± SD, kg/m^2^	31.43 ± 7.51	29.73 ± 7.36	< 0.001
Race/ethnicity (%)			
Mexican American	164 (12.8%)	1614 (13.8%)	< 0.001
Other Hispanic	157 (12.3%)	1233 (10.4%)	
Non-Hispanic white	612 (47.8%)	3997 (34.1%)	
Non-Hispanic black	208 (16.2%)	3029 (25.8%)	
Other race	140 (10.9%)	1869 (15.9%)	
Level of education (%)			
High school or below	592 (46.2%)	5477 (46.7%)	< 0.001
Some College	427 (33.3%)	3557 (30.3%)	
College or above	262 (20.5%)	2698 (23.9%)	
Poor income ratio (%)			
< 1.3	395 (30.8%)	3785 (32.3%)	< 0.001
1.3–3.49	539 (42.1%)	4607 (39.3%)	
≥ 3.5	347 (27.1%)	3340 (28.5%)	
Smoking behavior (%)			
Smoker	492 (38.4%)	3846 (32.8%)	< 0.001
Non-smoker	789 (61.6%)	7886 (67.2%)	
Alcohol consumption (%)			
Drinker	936 (73.1%)	8263 (70.4%)	< 0.001
Non-drinker	345 (26.9%)	3469 (29.6%)	
Hypertension (%)			
Yes	689 (53.8%)	4465 (38.1%)	< 0.001
No	592 (46.2%)	7267 (61.9%)	
Diabetes (%)			
Yes	329 (25.7%)	1752 (14.9%)	< 0.001
No	952 (74.3%)	9980 (85.1%)	
Coronary heart disease (%)			
Yes	103 (8.0%)	462 (3.9%)	< 0.001
No	1178 (92.0%)	11,270 (96.1%)	

Mean and standard deviation are used for continuous variables (e.g., age and body mass index), while proportions are used for categorical variables (e.g., gender and race/ethnicity). Chi-square test used to compare groups. p < 0.05 indicates significant difference

*P* p value, *SD* standard deviation

**Table 5:** Basic characteristics categorized by kidney stones (prostate cancer)

Characteristic	Kidney stones (n = 598)	Non-kidney stones (n = 4540)	P
Prostate cancer (%)			
Yes	40 (6.7%)	159 (3.5%)	< 0.001
No	558 (93.3%)	4381 (96.5%)	
Age, mean ± SD, year	58.57 ± 15.19	50.31 ± 17.89	< 0.001
Body mass index, mean ± SD, kg/m^2^	30.22 ± 6.6	29.16 ± 6.49	< 0.001
Race/ethnicity (%)			
Mexican American	61 (10.2%)	532 (11.7%)	< 0.001
Other Hispanic	63 (10.5%)	422 (9.3%)	
Non-Hispanic white	314 (52.5%)	1609 (35.4%)	
Non-Hispanic black	87 (14.5%)	1194 (26.3%)	
Other race	73 (12.2%)	783 (17.2%)	
Level of education (%)			
High school or below	259 (43.3%)	2133 (47.0%)	< 0.001
Some College	200 (33.4%)	1301 (28.7%)	
College or above	139 (23.2%)	1106 (24.4%)	
Poor income ratio (%)			
< 1.3	137 (22.9%)	1306 (28.8%)	< 0.001
1.3–3.49	268 (44.8%)	1814 (40.0%)	
≥ 3.5	193 (32.3%)	1420 (31.3%)	
Smoking behavior (%)			
Smoker	262 (43.8%)	2003 (44.1%)	< 0.001
Non-smoker	336 (56.2%)	2537 (55.9%)	
Alcohol consumption (%)			
Drinker	471 (78.8%)	3576 (78.8%)	< 0.001
Non-drinker	127 (21.2%)	964 (21.2%)	
Hypertension (%)			
Yes	320 (53.5%)	1636 (36.0%)	< 0.001
No	278 (46.5%)	2904 (64.0%)	
Diabetes (%)			
Yes	170 (28.4%)	700 (15.4%)	< 0.001
No	428 (71.6%)	3840 (84.6%)	
Coronary heart disease (%)			
Yes	72 (12.0%)	262 (5.8%)	< 0.001
No	526 (88.0%)	4278 (94.2%)	

Mean and standard deviation are used for continuous variables (e.g., age and body mass index), while proportions are used for categorical variables (e.g., gender and race/ethnicity). Chi-square test used to compare groups. p < 0.05 indicates significant difference

*P* p value, *SD* standard deviation

### Relationship between kidney stones and renal cell carcinoma, bladder cancer and prostate cancer

Table 6 presents the relationship between kidney stones and the risk of renal cell carcinoma, bladder cancer, and prostate cancer using three weighted multivariate logistic regression models. In the unadjusted Model 1, kidney stones were significantly associated with renal cell carcinoma (OR = 1.92, 95% CI 1.90–1.95, P < 0.001), bladder cancer (OR = 2.749, 95% CI 2.71–2.78, P < 0.001), and prostate cancer (OR = 2.03, 95% CI 2.02–2.04, P < 0.001). In Model 2 and Model 3, kidney stones remained significantly associated with all three types of urological cancers.

**Table 6:** Weighted multivariate logistic regression analyses between kidney stones and urological cancers

Risk of cancer	Kidney stones	Model 1	Model 2	Model 3
		OR(95% CI), P-value	OR(95% CI), P-value	OR(95% CI), P-value
Renal cell carcinoma	No	Ref	Ref	Ref
	Yes	1.924 (1.897, 1.951), < 0.001	1.205 (1.189, 1.223), < 0.001	1.014 (1.000,1.029), 0.06
Bladder cancer	No	Ref	Ref	Ref
	Yes	2.749 (2.714, 2.784), < 0.001	1.660(1.638, 1.682), < 0.001	1.603 (1.582, 1.624), < 0.001
Prostate cancer	No	Ref	Ref	Ref
	Yes	2.033 (2.022, 2.043), < 0.001	1.267 (1.260, 1.274), < 0.001	1.143 (1.137, 1.150), < 0.001

Model 1 is unjusted; Model 2 is adjusted for age, gender and race/ethnicity; Model 3 is adjusted for age, gender, race/ethnicity, education level, poor income ratio, body mass index, diabetes, hypertension, coronary heart disease,smoking behavior and alcohol consumption

*OR* Odd ratio, *CI* Confidence intervals

Additionally, this study also explored whether urological cancers increase the risk of kidney stones. Regardless of covariate adjustments, bladder cancer was associated with an increased risk of kidney stones. Notably, after controlling for all covariates, renal cell carcinoma (OR = 0.953, 95% CI 0.939–0.966, P < 0.001) and prostate cancer (OR = 0.984, 95% CI 0.979–0.989, P < 0.001) were inversely associated with the risk of kidney stones (Table 7).

**Table 7:** Weighted multivariate logistic regression analyses between cancers and kidney stones

Cancer	Model 1	Model 2	Model 3
	OR(95% CI), P-value	OR(95% CI), P-value	OR(95% CI), P-value
Non-renal cell carcinoma	Ref	Ref	Ref
Renal cell carcinoma	1.924 (1.897, 1.951), < 0.001	1.190 (1.173, 1.206), < 0.001	0.953 (0.939, 0.966), < 0.001
Non-bladder cancer	Ref	Ref	Ref
Bladder cancer	2.749 (2.714, 2.784), < 0.001	1.522(1.503, 1.542), < 0.001	1.485 (1.466, 1.505), < 0.001
Non-prostate cancer	Ref	Ref	Ref
Prostate cancer	2.033 (2.022, 2.043), < 0.001	1.036 (1.030, 1.042), < 0.001	0.984 (0.979, 0.989), < 0.001

Model 1 is unjusted; Model 2 is adjusted for age, gender and race/ethnicity; Model 3 is adjusted for age, gender, race/ethnicity, education level, poor income ratio, body mass index, diabetes, hypertension, coronary heart disease,smoking behavior and alcohol consumption

*OR* Odd ratio, *CI* Confidence intervals

Subgroup analysis in Table 8 showed that, regardless of covariate adjustments, females with kidney stones had a higher risk of renal cell carcinoma and bladder cancer than males. Although some age-stratified subgroups lacked cancer cases, older individuals with kidney stones were at an increased risk of urological cancers. Stratification by household income revealed that individuals with kidney stones in higher-income groups had a lower risk of renal cell carcinoma, bladder cancer, and prostate cancer. Additionally, higher education levels were associated with an increased risk of urological cancers among kidney stones patients. Tables 9, 10 and 11 present the subgroup analysis of kidney stones risk among different cancer patients.

**Table 8:** Stratified analysis between kidney stones and urological cancers

Stratification	Kidney stones	Renal cell carcinoma			Bladder cancer			Prostate cancer		
		Model 1	Model 2	Model 3	Model 1	Model 2	Model 3	Model 1	Model 2	Model 3
		OR (95% CI), P-value	OR (95% CI), P-value	OR (95% CI), P-value	OR (95% CI), P-value	OR (95% CI), P-value	OR (95% CI), P-value	OR (95% CI), P-value	OR (95% CI), P-value	OR (95% CI), P-value
Male	No	Ref	Ref	Ref	Ref	Ref	Ref	–	–	–
	Yes	1.247 (1.225, 1.270), < 0.001	0.081 (0.790, 0.820), < 0.001	0.655 (0.643, 0.668), < 0.001	2.294 (2.260, 2.330), < 0.001	1.385 (1.364, 1.406), < 0.001	1.448 (1.425, 1.471), < 0.001	–	–	–
Female	No	Ref	Ref	Ref	Ref	Ref	Ref	–	–	–
	Yes	3.416( 3.341, 3.492), < 0.001	3.101 (3.033, 3.170), < 0.001	2.843 (2.779, 2.908), < 0.001	3.094 (3.021, 3.169), < 0.001	2.888(2.820, 2.958), < 0.001	2.161 (2.108, 2.215), < 0.001	–	–	–
20–39	No	–	–	–	–	–	–	–	–	–
	Yes	–	–	–	–	–	–	–	–	–
40–59	No	Ref	Ref	Ref	–	–	–	Ref	Ref	Ref
	Yes	1.010 (0.971, 1.050), 0.63	0.798 (0.767, 0.829), < 0.001	0.672 (0.645–0.699), < 0.001	–	–	–	1.131 (1.102–1.160), < 0.001	0.824 (0.803, 0.846), < 0.001	1.819 (1.767, 1.872), < 0.001
60–80	No	Ref	Ref	Ref	Ref	Ref	Ref	Ref	Ref	Ref
	Yes	1.749 (1.722, 1.775), < 0.001	1.402 (1.381, 1.424), < 0.001	1.111 (1.094, 1.129), < 0.001	2.133 (2.106, 2.161), < 0.001	1.656 (1.635, 1.678), < 0.001	1.614 (1.592–1.635), < 0.001	1.277 (1.270–1.285), < 0.001	1.267 (1.260, 1.275), < 0.001	1.167 (1.160, 1.174), < 0.001
Mexican American	No	Ref	Ref	Ref	–	–	–	Ref	Ref	Ref
	Yes	4.159 (4.019, 4.304), < 0.001	2.709 (2.616, 2.805), < 0.001	1.995 (1.915, 2.078), < 0.001	–	–	–	6.558 (6.300, 6.827), < 0.001	3.106 (2.975, 3.243), < 0.001	2.428 (2.314–2.548), < 0.001
Other Hispanic	No	Ref	Ref	Ref	–	–	–	Ref	Ref	Ref
	Yes	3.634 (3.442, 3.857), < 0.001	2.067 (1.951, 2.191), < 0.001	1.186E + 172 (5.256E + 93, 2.678E + 250), < 0.001	–	–	–	1.423(1.383, 1.465), < 0.001	0.999(0.970, 1.030), 0.963	0.472(0.454–0.491), < 0.001
Non-Hispanic White	No	Ref	Ref	Ref	Ref	Ref	Ref	Ref	Ref	Ref
	Yes	1.242 (1.219, 1.265), < 0.001	0.838 (0.823, 0.854), < 0.001	0.751 (0.737, 0.765), < 0.001	2.481 (2.448, 2.515), < 0.01	1.578 (1.557, 1.600), < 0.001	1.516 (1.495, 1.537), < 0.001	1.994 (1.982, 2.005), < 0.001	1.289 (1.281, 1.296), < 0.001	1.194 (1.186, 1.201), < 0.001
Non-Hispanic Black	No	Ref	Ref	Ref	–	–	–	Ref	Ref	Ref
	Yes	3.685 (3.539, 3.837), < 0.001	2.714 (2.606, 2.827), < 0.001	2.305 (2.211, 2.402), < 0.001	–	–	–	2.758 (2.710, 2.807), < 0.001	2.082 (2.042, 2.122), < 0.001	1.807 (1.771, 1.845), < 0.001
Other Rance-Including Multi-Racial	No	Ref	Ref	Ref	Ref	Ref	Ref	–	–	–
	Yes	13.453 (12.570, 14.399), < 0.001	11.881 (11.090, 12.729), < 0.001	23.258 (23.582, 29.239), < 0.001	9.004 (8.490, 9.549), < 0.001	6.262 (5.901, 6.645), < 0.001	6.954 (6.453, 7.495), < 0.001	–	–	–
High school graduate or below	No	Ref	Ref	Ref	Ref	Ref	Ref	Ref	Ref	Ref
	Yes	1.543 (1.504, 1.584), < 0.001	1.350 (1.316, 1.386), < 0.001	1.249 (1.217–1.282), < 0.001	1.816 (1.771, 1.862), < 0.001	1.304 (1.271, 1.338), < 0.001	1.366 (1.331–1.402), < 0.001	2.135 (2.115, 2.156), < 0.001	1.554 (1.539, 1.570), < 0.001	1.506 (1.491, 1.521), < 0.001
Some college	No	Ref	Ref	Ref	Ref	Ref	Ref	Ref	Ref	Ref
	Yes	3.354 (3.295, 3.414), < 0.001	1.842 (1.809, 1.876), < 0.001	2.015 (1.978–2.053), < 0.001	2.078 (2.037, 2.120), < 0.001	1.353 (1.326, 1.381), < 0.001	1.145 (1.121–1.169), < 0.001	1.756 (1.739, 1.773), < 0.001	1.043 (1.032, 1.053), < 0.001	1.048 (1.037, 1.059), < 0.001
College graduate or above	No	–	–	–	Ref	Ref	Ref	Ref	Ref	Ref
	Yes	–	–	–	7.347 (7.167, 7.532), < 0.001	3.439 (3.351, 3,529), < 0.001	4.073 (3.958, 4.191), < 0.001	2.090 (2.073, 2.108), < 0.001	1.100 (1.091, 1.110), < 0.001	0.866 (0.857, 0.874), < 0.001
PIR < 1.3	No	Ref	Ref	Ref	Ref	Ref	Ref	Ref	Ref	Ref
	Yes	3.693 (3.543, 3.849), < 0.001	3.495 (3.352, 3.644), < 0.001	4.590 (4.400, 4.789), < 0.001	4.888 (4.755, 5.025), < 0.001	3.016 (2.931, 3.103), < 0.001	4.051 (3.925, 4.182), < 0.001	7.691 (7.574, 7.809), < 0.001	4.464 (4.391, 4.538), < 0.001	4.688 (4.606, 4.770), < 0.001
1.3 < PIR < 3.49	No	Ref	Ref	Ref	Ref	Ref	Ref	Ref	Ref	Ref
	Yes	2.492 (2.447, 2.538), < 0.001	1.819 (1.785, 1.853), < 0.001	1.430 (1.404, 1.457), < 0.001	2.608 (2.566, 2.652), < 0.001	1.710 (1.682, 1.739), < 0.001	1.504 (1.478, 1.530), < 0.001	2.255 (2.236, 2.273), < 0.001	1.511 (1.498, 1.524), < 0.001	1.347 (1.335, 1.359), < 0.001
PIR ≥ 3.5	No	Ref	Ref	Ref	Ref	Ref	Ref	Ref	Ref	Ref
	Yes	0.984 (0.958, 1.012), 0.257	0,462 (0.449, 0.475), < 0.001	0.417 (0.406, 0.429), < 0.001	1.645 (1.592, 1.699), < 0.001	1.015 (0.982, 1.049), 0.386	0.902 (0.871, 0.933), < 0.001	1.330 (1.319, 1.340), < 0.001	0.815 (0.808, 0.822), < 0.001	0.754 (0.748, 0.761), < 0.001

Model 1 is unjusted; Model 2 is adjusted for age, gender and race/ethnicity; Model 3 is adjusted for age, gender, race/ethnicity, education level, PIR, BMI, diabetes, hypertension, coronary heart disease,smoking behavior and alcohol consumption; The missing data in the table is due to the absence of cancer patients in that stratification group

*BMI* body mass index, *CI* Confidence intervals, *OR* Odd ratio, *PIR* poor income ration

**Table 9:** Stratified analysis between renal cell carcinoma and kidney stones

Stratification	Renal cell carcinoma	The risk of kidney stones		
		Model 1	Model 2	Model 3
		OR(95% CI), P-value	OR(95% CI), P-value	OR(95% CI), P-value
Male	No	Ref	Ref	Ref
	Yes	1.247 (1.225, 1.270), < 0.001	0.710 (0.697, 0.723), < 0.001	0.542 (0.532, 0.552), < 0.001
Female	No	Ref	Ref	Ref
	Yes	3.416 (3.341, 3.492), < 0.001	2.692 (2.633, 2.752), < 0.001	2.165 (2.117, 2.215), < 0.001
20–39	No	–	–	–
	Yes	–	–	–
40–59	No	Ref	Ref	Ref
	Yes	1.010 (0.971, 1.050), 0.63	0.790 (0.760, 0.822), < 0.001	0.733 (0.704, 0.762), < 0.001
60–80	No	Ref	Ref	Ref
	Yes	1.749 (1.722, 1.775), < 0.001	1.431 (1.409, 1.453), < 0.001	1.157 (1.139, 1.175), < 0.001
Mexican American	No	Ref	Ref	Ref
	Yes	4.159 (4.019, 4.304), < 0.001	2.520 (2.433, 2.610), < 0.001	1.466 (1.414, 1.519), < 0.001
Other Hispanic	No	Ref	Ref	Ref
	Yes	3.643 (3.442, 3.857), < 0.001	1.918 (1.811, 2.030), < 0.001	0.556 (0.524, 0.591), < 0.001
Non-Hispanic White	No	Ref	Ref	Ref
	Yes	1.242 (1.219, 1.265), < 0.001	0.832(0.816, 0.847), < 0.001	0.663 (0.650, 0.675), < 0.001
Non-Hispanic Black	No	Ref	Ref	Ref
	Yes	3.685 (3.539, 3.837), < 0.001	2.565 (2.463, 2.672), < 0.001	2.133 (2.047, 2.223), < 0.001
Other Rance-Including Multi-Racial	No	Ref	Ref	Ref
	Yes	13.453 (12.570, 14.399), < 0.001	10.037 (9.373, 10.747), < 0.001	5.271 (4.920, 5.647), < 0.001
High school graduate or below	No	Ref	Ref	Ref
	Yes	1.543 (1.504, 1.584), < 0.001	1.229 (1.198, 1.262), < 0.001	1.106 (1.077, 1.135), < 0.001
Some college	No	Ref	Ref	Ref
	Yes	3.354 (3.295, 3.414), < 0.001	1.918 (1.884, 1.953), < 0.001	1.496 (1.469, 1.523), < 0.001
College graduate or above	No	–	–	–
	Yes	–	–	–
PIR < 1.3	No	Ref	Ref	Ref
	Yes	3.693 (3.543, 3.849), < 0.001	2.740 (2.629, 2.856), < 0.001	3.618 (3.469, 3.773), < 0.001
1.3 < PIR < 3.49	No	Ref	Ref	Ref
	Yes	2.492 (2.447, 2.538), < 0.001	1.721 (1.690, 1.753), < 0.001	1.269 (1.245, 1.293), < 0.001
PIR ≥ 3.5	No	Ref	Ref	Ref
	Yes	0.984 (0.958, 1.012), 0.26	0.457 (0.444, 0.469), < 0.001	0.366 (0.356, 0.376), < 0.001

Model 1 is unjusted; Model 2 is adjusted for age, gender and race/ethnicity; Model 3 is adjusted for age, gender, race/ethnicity, education level, PIR, BMI, diabetes, hypertension, coronary heart disease,smoking behavior and alcohol consumption. The missing data in the table is due to the absence of kidney stone patients in that stratification group

*BMI* body mass index, *CI* Confidence intervals, *OR* Odd ratio, *PIR* poor income ration

**Table 10:** Stratified analysis between bladder cancer and kidney stones

Stratification	Bladder cancer	The risk of kidney stones		
		Model 1	Model 2	Model 3
		OR (95% CI), P-value	OR (95% CI), P-value	OR (95% CI), P-value
Male	No	Ref	Ref	Ref
	Yes	2.294(2.260, 2.330), < 0.001	1.078(1.061, 1.094), < 0.001	1.147(1.129, 1.165), < 0.001
Female	No	Ref	Ref	Ref
	Yes	3.094(3.021, 3.169), < 0.001	2.398(2.341, 2.456), < 0.001	2.091(2.041, 2.142), < 0.001
20–39	No	–	–	–
	Yes	–	–	–
40–59	No	–	–	–
	Yes	–	–	–
60–80	No	Ref	Ref	Ref
	Yes	2.133(2.106, 2.161), < 0.001	1.715(1.693, 1.737), < 0.001	1.694(1.673, 1.717), < 0.001
Mexican American	No	–	–	–
	Yes	–	–	–
Other Hispanic	No	–	–	–
	Yes	–	–	–
Non-Hispanic White	No	Ref	Ref	Ref
	Yes	2.481(2.448, 2.515), < 0.001	1.491(1.471, 1.511), < 0.001	1.378(1.359, 1.397), < 0.001
Non-Hispanic Black	No	–	–	–
	Yes	–	–	–
Other Rance-Including Multi-Racial	No	Ref	Ref	Ref
	Yes	9.004(8.490, 9.549), < 0.001	5.027(4.739, 5.333), < 0.001	3.621(3.407, 3.848), < 0.001
High school graduate or below	No	Ref	Ref	Ref
	Yes	1.816(1.771, 1.862), < 0.001	1.185(1.155, 1.215), < 0.001	1.262(1.231, 1.295), < 0.001
Some college	No	Ref	Ref	Ref
	Yes	2.078(2.037, 2.120), < 0.001	1.182(1.159, 1.206), < 0.001	1.072(1.050, 1.094), < 0.001
College graduate or above	No	Ref	Ref	Ref
	Yes	7.347(7.167, 7.532), < 0.001	2.987(2.913, 3.064), < 0.001	2.229(2.173, 2.287), < 0.001
PIR < 1.3	No	Ref	Ref	Ref
	Yes	4.888(4.755, 5.025), < 0.001	3.537(3.440, 3.637), < 0.001	3.821(3.714, 3.930), < 0.001
1.3 < PIR < 3.49	No	Ref	Ref	Ref
	Yes	2.608(2.566, 2.652), < 0.001	1.552(1.527, 1.578), < 0.001	1.262(1.240, 1.283), < 0.001
PIR ≥ 3.5	No	Ref	Ref	Ref
	Yes	1.645(1.592, 1.699), < 0.001	0.827(0.800, 0.854), < 0.001	0.953(0.922, 0.985), < 0.001

Model 1 is unjusted; Model 2 is adjusted for age, gender and race/ethnicity; Model 3 is adjusted for age, gender, race/ethnicity, education level, PIR, BMI, diabetes, hypertension, coronary heart disease,smoking behavior and alcohol consumption. The missing data in the table is due to the absence of kidney stone patients in that stratification group

*BMI* body mass index, *CI* Confidence intervals, *OR* Odd ratio, *PIR* poor income ration

**Table 11:** Stratified analysis between prostate cancer and kidney stones

Stratification	Prostate cancer	The risk of kidney stones		
		Model 1	Model 2	Model 3
		OR (95% CI), P-value	OR (95% CI), P-value	OR (95% CI), P-value
20–39	No	–	–	–
	Yes	–	–	–
40–59	No	Ref	Ref	Ref
	Yes	1.131 (1.102, 1.160), < 0.001	0.941 (0.918, 0.966), < 0.001	0.825 (0.804, 0.846), < 0.001
60–80	No	Ref	Ref	Ref
	Yes	1.277 (1.270, 1.285), < 0.001	1.272 (1.264, 1.279), < 0.001	1.232 (1.225, 1.239), < 0.001
Mexican American	No	Ref	Ref	Ref
	Yes	6.558 (6.300, 6.827), < 0.001	2.121 (2.036, 2.210), < 0.001	1.923 (1.843–2.006), < 0.001
Other Hispanic	No	Ref	Ref	Ref
	Yes	1.423 (1.383, 1.465), < 0.001	1.021 (0.991, 1.051), 0.17	0.815 (0.790, 0.841), < 0.001
Non-Hispanic White	No	Ref	Ref	Ref
	yes	1.994 (1.982, 2.005), < 0.001	1.065 (1.059, 1.072), < 0.001	1.030 (1.023, 1.036), < 0.001
Non-Hispanic Black	No	Ref	Ref	Ref
	Yes	2.758(2.710, 2.807), < 0.001	1.564(1.536, 1.594), < 0.001	1.374 (1.349, 1.400), < 0.001
Other Race-Including Multi-Racial	No	–	–	–
	Yes	–	–	–
High school graduate or below	No	Ref	Ref	Ref
	yes	2.135 (2.115, 2.156), < 0.001	1.181 (1.169, 1.192), < 0.001	1.198 (1.186, 1.210), < 0.001
Some college	No	Ref	Ref	Ref
	Yes	1.756 (1.739, 1.773), < 0.001	0.836 (0.828, 0.845), < 0.001	0.852 (0.843, 0.860), < 0.001
College graduate or above	No	Ref	Ref	Ref
	Yes	2.090 (2.073, 2.108), < 0.001	1.005 (0.996, 1.013), 0.30	0.821 (0.814, 0.829), < 0.001
PIR < 1.3	No	Ref	Ref	Ref
	Yes	7.691 (7.574, 7.809), < 0.001	3.972 (3.910, 4.036), < 0.001	3.963 (3.897, 4.030), < 0.001
1.3 < PIR < 3.49	No	Ref	Ref	Ref
	Yes	2.255 (2.236, 2.273), < 0.001	1.134 (1.124, 1.143), < 0.001	1.037 (1.028, 1.046), < 0.001
PIR ≥ 3.5	No	Ref	Ref	Ref
	Yes	1.330 (1.319, 1.340), < 0.001	0.700 (0.694, 0.706), < 0.001	0.653 (0.648, 0.658), < 0.001

Model 1 is unjusted; Model 2 is adjusted for age, gender and race/ethnicity; Model 3 is adjusted for age, gender, race/ethnicity, education level, PIR, BMI, diabetes, hypertension, coronary heart disease,smoking behavior and alcohol consumption. The missing data in the table is due to the absence of kidney stone patients in that stratification group

*BMI* body mass index, *CI* Confidence intervals, *OR* Odd ratio, *PIR* poor income ration

### Causal effects of kidney stones disease on urological cancers

**Fig. 3:**
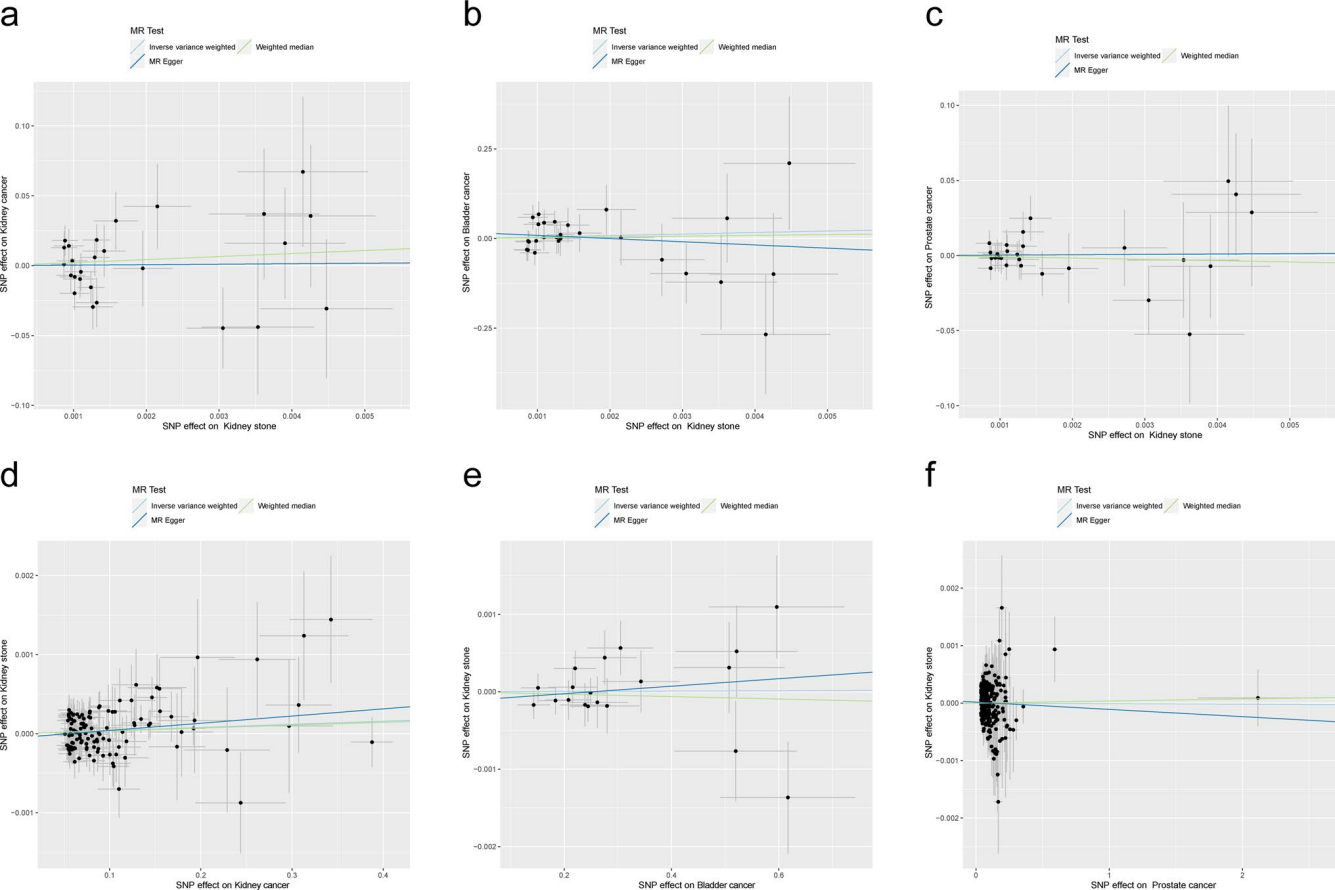
Causal effect between kidney stones and urological cancers. The scatter plots present the genetic relationship between kidney stones and three types of urological cancers. The analysis was conducted using the IVW method, weighted median method, and MR-Egger method. The slope of each line represents the estimated MR effect obtained from the corresponding method. **A**–**C** are plots of the effect size of each SNP of kidney stone on the risk of renal cell carcinoma (**A**), bladder cancer (**B**) and prostate cancer (**C**). **D**–**F** are plots of the effect size of each SNP of renal cell carcinoma (**D**), bladder cancer (**E**) and prostate cancer (**F**) on kidney stone risk

After screening, a total of 30 SNPs associated with kidney stones risk were included. The results of the MR analysis, using IVW method, indicated that genetically predicted kidney stones were not significantly causally associated to renal cell carcinoma (OR = 1.41, 95% CI 0.01–219.77, P = 0.893), bladder cancer (OR = 64.47, 95% CI 0.00–1.94 × 10^7^, P = 0.517), or prostate cancer (OR = 1.31, 95% CI 0.04–43.84, P = 0.879). The results from the weighted median method and MR-Egger method were consistent with these findings (Fig. 3 A–C and Table 12).

**Table 12:** MR analysis for causal relationship between kidney stones and urological cancers

Exposure	Outcome	Method	nSNP	OR (95% CI)	P
Kidney stones	Renal cell carcinoma	Inverse variance weighted	27	1.41 (0.001, 219.77)	0.89
		Weighted median	27	8.72 (0.01, 8166.15)	0.54
		MR Egger	27	1.41 (0.00, 375,325.77)	0.96
Kidney stones	Bladder cancer	Inverse variance weighted	27	64.47 (0.00, 1.94E + 07)	0.52
		Weighted median	27	9.00 (0.00, 6.99E + 08)	0.81
		MR Egger	27	0.00 (0.00, 5.10E + 10)	0.61
Kidney stones	Prostate cancer	Inverse variance weighted	28	1.31 (0.04, 43.84)	0.88
		Weighted median	28	0.43 (0.00, 58.52)	0.73
		MR Egger	28	1.26 (0.00, 10,179.25)	0.96
Renal cell carcinoma	Kidney stones	Inverse variance weighted	119	1.00 (1.00, 1.00)	0.08
		Weighted median	119	1.00 (1.00, 1.00)	0.32
		MR Egger	119	1.00 (1.00, 1.00)	0.10
Bladder cancer	Kidney stones	Inverse variance weighted	19	1.00 (1.00, 1.00)	0.94
		Weighted median	19	1.00 (1.00, 1.00)	0.66
		MR Egger	19	1.00 (1.00, 1.00)	0.51
Prostate cancer	Kidney stones	Inverse variance weighted	296	1.00 (1.00, 1.00)	0.94
		Weighted median	296	1.00 (1.00, 1.00)	0.88
		MR Egger	296	1.00 (1.00, 1.00)	0.52

*nSNP* number of single nucleotide polymorphism, *OR* odd ratio, *P* p-value

To further explore the potential causal relationship between a history of urological cancers and the risk of kidney stones, reverse MR analysis was conducted. Using the IVW method, the ORs for the impact of renal cell carcinoma, bladder cancer, and prostate cancer on the risk of kidney stones were 1.00 (95% CI 1.00–1.00, P = 0.082), 1.00 (95% CI 1.00–1.00, P = 0.939), and 1.00 (95% CI 1.00–1.00, P = 0.943), respectively (Fig. 3 D–F and Table 12).

**Fig. 4:**
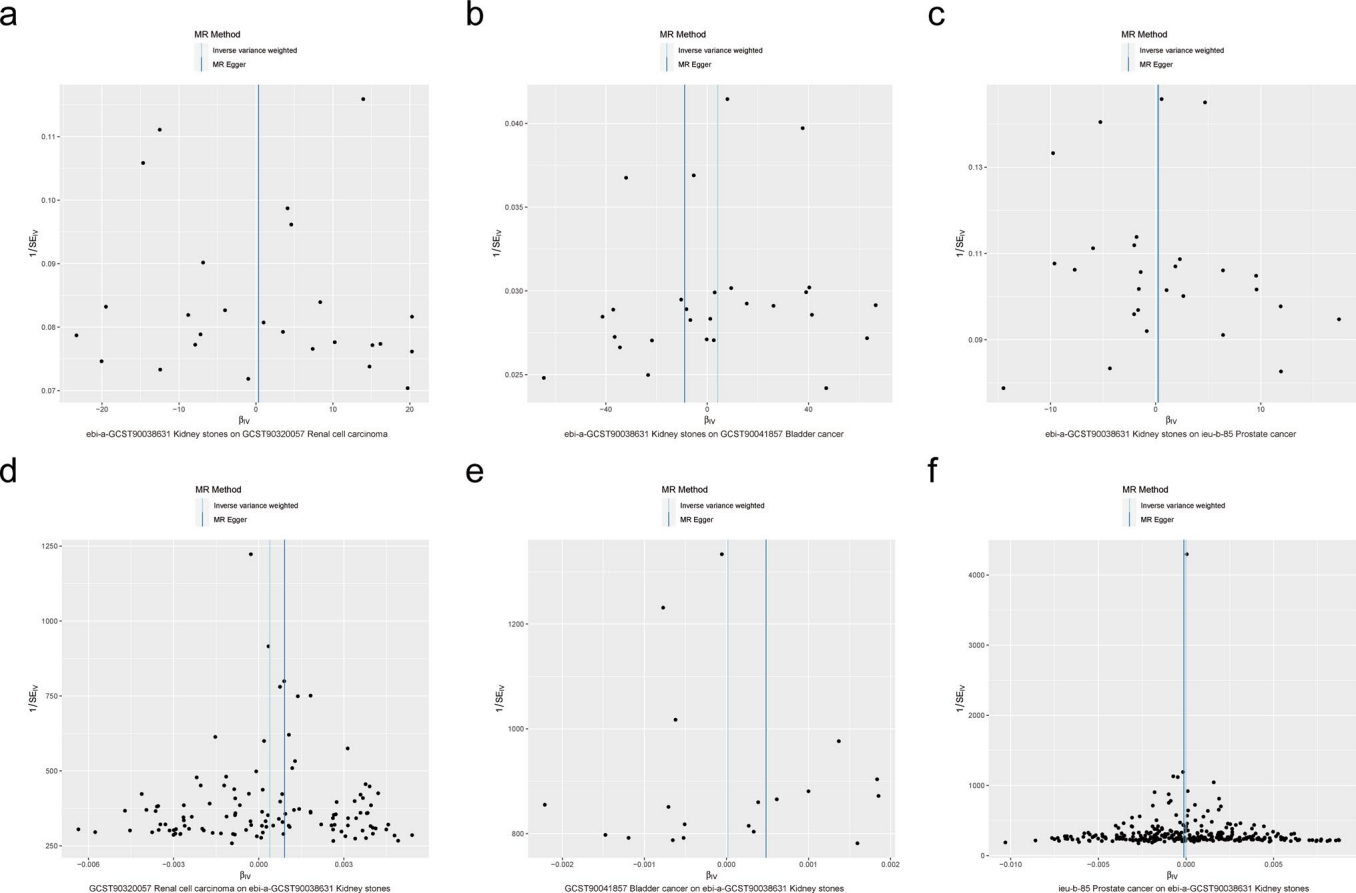
The funnel plots of heterogeneity analysis between kidney stones and urological cancers. **A**–**C** are funnel plots of forward MR analysis between kidney stones and renal cell carcinoma (**A**), bladder cancer (**B**) and prostate cancer (**C**). **E**, **F** are funnel plots of reverse MR. The funnel plots illustrate the effect estimates of each IV on the exposure and outcome. The x-axis represents the effect estimates of the IVs on the exposure, while the y-axis denotes their effect estimates on the outcome. A symmetric distribution indicates low heterogeneity

**Fig. 5:**
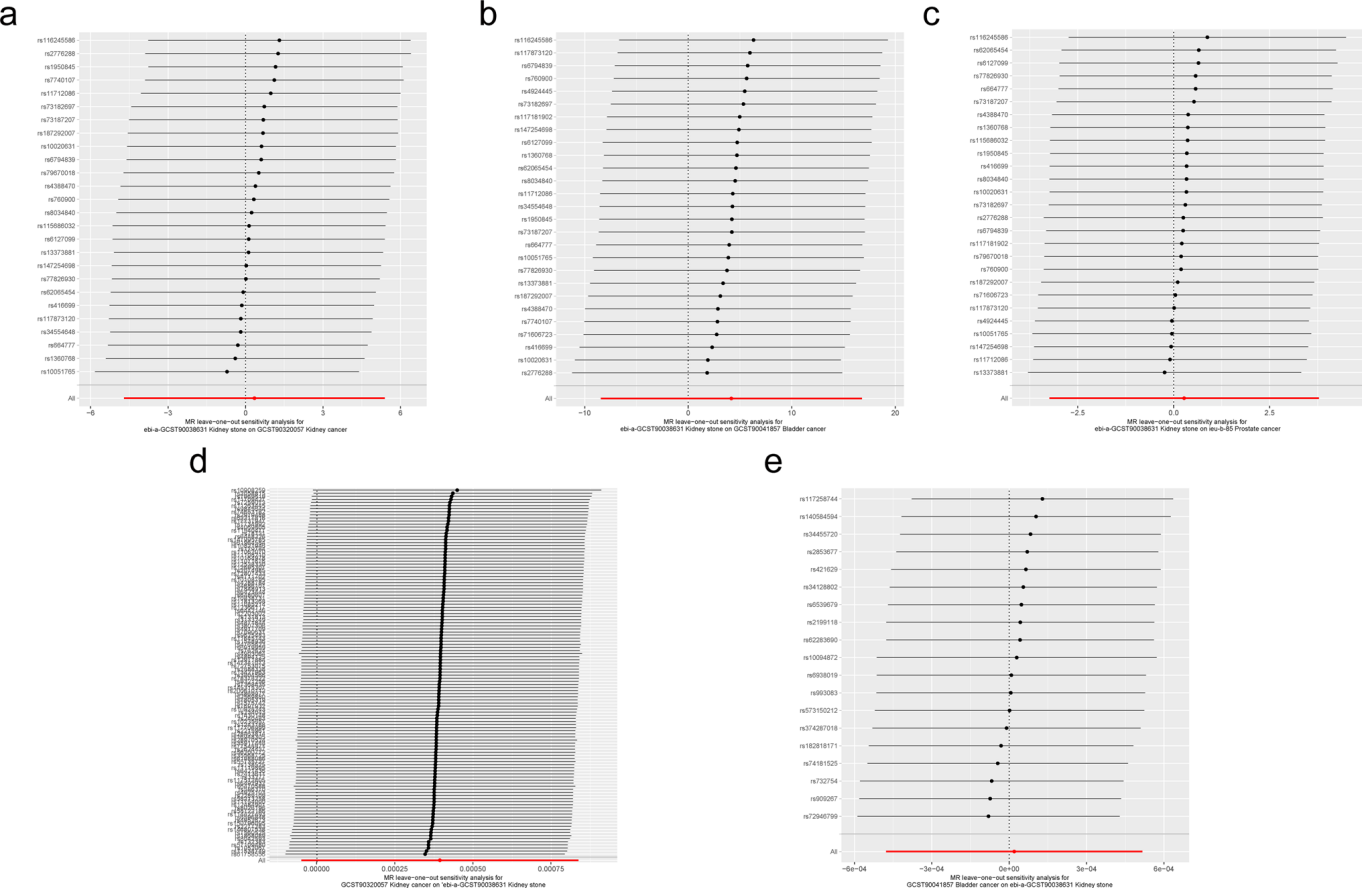
The leave-one-out analysis results between kidney stones and urological cancers. **A**–**C** are plots of LOO analysis of forward MR analysis between kidney stone and renal cell carcinoma (**A**), bladder cancer (**B**) and prostate cancer (**C**). **D**, **E** are plots of LOO analysis of reverse MR. The x-axis represents the estimated causal effect after excluding each specific SNP, while the y-axis denotes the SNP identifier. The LOO results for all SNPs intersect the vertical line and align with the red horizontal line, indicating that no single SNP significantly disrupts the MR results

As a result of the sensitivity analysis, no evidence of heterogeneity or pleiotropy was found among the selected IVs in this study (Fig. 4 and Table 13). The LOO analysis did not identify any single SNP that could alter the overall results (Fig. 5 and Supplementary Fig. 1), suggesting the robustness of the initial findings.

**Table 13:** Sensitivity analysis of forward and reverse MR analysis

Exposure	Outcome	nSNP	Heterogeneity				Pleiotropy test		
			Method	Q	df	P-value	Egger_intercept	P-value	Global test’s p-value of MR-PRESSO
Kidney stones	Renal cell carcinoma	27	MR Egger	30.77	24	0.16	5.74E-06	1.00	0.89
			Inverse variance weighted	30.77	25	0.20			
Kidney stones	Bladder cancer	27	MR Egger	23.55	25	0.55	1.76E-02	0.42	0.51
			Inverse variance weighted	24.23	26	0.56			
Kidney stones	Prostate cancer	28	MR Egger	14.53	25	0.95	5.67E-05	0.99	0.84
			Inverse variance weighted	14.53	26	0.97			
Renal cell carcinoma	Kidney stones	119	MR Egger	103.67	117	0.81	− 5.01E-05	0.30	0.07
			Inverse variance weighted	104.76	118	0.80			
Bladder cancer	Kidney stones	19	MR Egger	17.65	17	0.41	− 1.23E-04	0.51	0.94
			Inverse variance weighted	18.13	18	0.45			
Prostate cancer	Kidney stones	296	MR Egger	258.50	294	0.93	1.62E-05	0.40	0.94
			Inverse variance weighted	259.20	295	0.93			

Heterogeneity was assessed using Cochran’s Q test within the Inverse-variance weighted method and MR-Egger method. Pleiotropy was examined using MR-Egger regression and the global test in MR-PRESSO

*df* degrees of freedom, *MR-PRESSO* Mendelian Randomization Pleiotropy Residual Sum and Outlier, *Q* Cochran’s Q statistic

## Discussion

Using a large-scale cross-sectional dataset and GWAS, we provide novel insights into the complex relationships between kidney stones and three common urological cancers including renal cell carcinoma, bladder cancer, and prostate cancer. After comprehensively adjusting for age, sex, race, and other potential confounders, our findings suggest that kidney stones are associated with an increased risk of renal cell carcinoma, bladder cancer, and prostate cancer. Notably, these three urological cancers are also found to increase the risk of kidney stones. However, MR analyses did not support a causal relationship between kidney stones and these urological cancers. Sensitivity analyses and the robust effects of genetic instruments further confirmed the reliability of our findings.

The association between kidney stones and various cancers has gained increasing attention. Chia-Jen Shih conducted an analysis using data from Taiwan’s National Health Insurance Research Database and discovered that, in addition to elevating the risk of renal cell carcinoma and bladder cancer, patients with kidney stones were also found to have an increased risk of thyroid cancer, hematologic malignancies, breast cancer, lung cancer, and digestive system cancers [12]. A study by Hemminki et al. identified a weak association between parental kidney stone history and the development of salivary gland and small intestinal neoplasms in their offspring [33]. As early as 1997, Chow et al. discovered that patients hospitalized for kidney stones had a higher risk of renal or ureteral cancer, and the location of the stones often coincided with the site of the tumor. Moreover, patients hospitalized for bladder stones had nearly a three-fold increased risk of bladder cancer [14]. These findings strongly suggested correlations between kidney stones and urological cancers. Our study found that kidney stones were significantly associated with an increased risk of renal cell carcinoma, bladder cancer, and prostate cancer, after adjusting for covariates, which is consistent with previous findings [9–13].

While the mechanisms underlying the observed associations between kidney stones disease and renal cell carcinoma, bladder cancer, and prostate cancer remain unclear, several plausible hypotheses have been proposed. The chronic presence of kidney stones may continuously stimulate local tissues, inducing inflammation and promoting the secretion of cytokines and chemokines by inflammatory cells [12, 34]. During acute inflammation, elevated levels of tumor necrosis factor-α, interleukin-1, and interleukin-6 may induce abnormal cell proliferation and malignant tumor progression [35, 36]. Moreover, chronic stimulation and inflammation caused by kidney stones can lead to squamous metaplasia of the renal collecting system, which may eventually progress to squamous cell carcinoma [37–40]. In recent years, dysbiosis of the gut microbiota has been observed in patients with kidney stones, characterized by a significant increase in pro-inflammatory bacteria and a decrease in anti-inflammatory bacteria [41, 42]. Alterations in the gut microbiota have been correlated to an increased risk of various cancers, potentially mediated by changes in the local intestinal immune system [43–45]. A MR analysis has shown a causal relationship between the gut microbiota and urological cancers [46]. Although the specific mechanisms remain unclear, this may partially explain the association between kidney stones and renal cell carcinoma, bladder cancer and prostate cancer.

Interestingly, our results indicated an increased risk of prostate cancer among patients with kidney stones, consistent with the findings of a previous study [47]. A study based on the Taiwan Longitudinal Health Insurance Database 2000 found a significantly higher prevalence of kidney stones among 2,900 prostate cancer patients aged over 40 compared to a non-prostate cancer cohort [16]. Although our findings provide stronger evidence to support this association, there is still a paucity of research on the relationship between kidney stones and prostate cancer. Further studies are warranted to elucidate the underlying mechanisms linking these two diseases.

Of note, our findings from the cross-sectional analysis suggest an increased risk of kidney stones among patients with a history of bladder cancer. However, there is a lack of epidemiological or clinical studies corroborating this finding. Furthermore, due to the absence of detailed clinical information within the NHANES database, the potential influence of cancer treatment on the kidney stones incidence and the possibility of surveillance bias resulting from increased medical monitoring cannot be excluded [48–50]. Therefore, this conclusion should be interpreted with caution.

Our study has several strengths. First, the NHANES database provided a large sample size, which enhanced the statistical power of our study. Second, to overcome the limitation of cross-sectional studies in establishing causality, we complemented our approach with MR analysis. However, the conclusions drawn from the MR analysis and the NHANES data were not entirely consistent. Cross-sectional data capture the status at a single point in time. In contrast, MR analysis focuses on the long-term causal effects of an exposure on an outcome, which may account for the differences in results. Given the findings of prior researches, the result from MR analysis, which may be influenced by the sample size and the statistical power of the SNPs, was unable to definitively rule out a potential causal relationship between kidney stones and renal cell carcinoma, bladder cancer, and prostate cancer.

There are several limitations to this study. First, in the cross-sectional study, information on the history of kidney stones and cancers was obtained through questionnaires, which lacked clear temporal information regarding the onset of kidney stones and cancers. Second, due to the lack of data on the composition of kidney stones, we were unable to further analyze the impact of different types of stones on urological cancer risk. Third, NHANES data is derived from the American population, whereas the MR study primarily focuses on European individuals. This potential ethnic heterogeneity may lead to inconsistencies in the conclusions.

## Conclusions

Observational studies have consistently demonstrated a significant association between kidney stones and an increased risk of renal cell carcinoma, bladder cancer, and prostate cancer. MR analysis supports no causal relationship between kidney stones and renal cell carcinoma, bladder cancer and prostate cancer. Future studies with larger sample sizes and more robust genetic instruments are needed to definitively assess the causal relationship of this association.

## Supplementary Information

Supplementary material 1: Fig. 1 The leave-one-out analysis results between prostate cancer and kidney stone. The x-axis represents the estimated causal effect after excluding each specific SNP, while the y-axis denotes the SNP identifier. The red horizontal line represents the estimated overall causal effect derived from all SNPs. The LOO results for all SNPs intersect the vertical line and align with the red horizontal line, indicating that no single SNP significantly disrupts the MR results.

## Data Availability

Data collected and analyzed in this study are available at NHANES website: https://www.cdc.gov/nchs/nhanes/. The remaining data from the study can be found in the article and supplementary materials. Other data supporting the findings of this study are available from the corresponding author upon reasonable request.
