# Supplementary material for: Association between kidney stones and urological cancers: results from the NHANES 2007–2020 and Mendelian randomization study
Source: Discov Oncol. 2025 Apr 24;16:601. doi: 10.1007/s12672-025-02415-4 (PMC12021769; doi:10.1007/s12672-025-02415-4)
Supplement: Supplementary file 1 — Supplementary material 1: Fig. 1 The leave-one-out analysis results between prostate cancer and kidney stone. The x-axis represents the estimated causal effect after excluding each specific SNP, while the y-axis denotes the SNP identifier. The red horizontal line represents the estimated overall causal effect derived from all SNPs. The LOO results for all SNPs intersect the vertical line and align with the red horizontal line, indicating that no single SNP significantly disrupts the MR results. [file 12672_2025_2415_MOESM1_ESM.docx]

**Supplementary Information**

**Supplementary Fig.1** The leave-one-out analysis results between prostate cancer and kidney stone


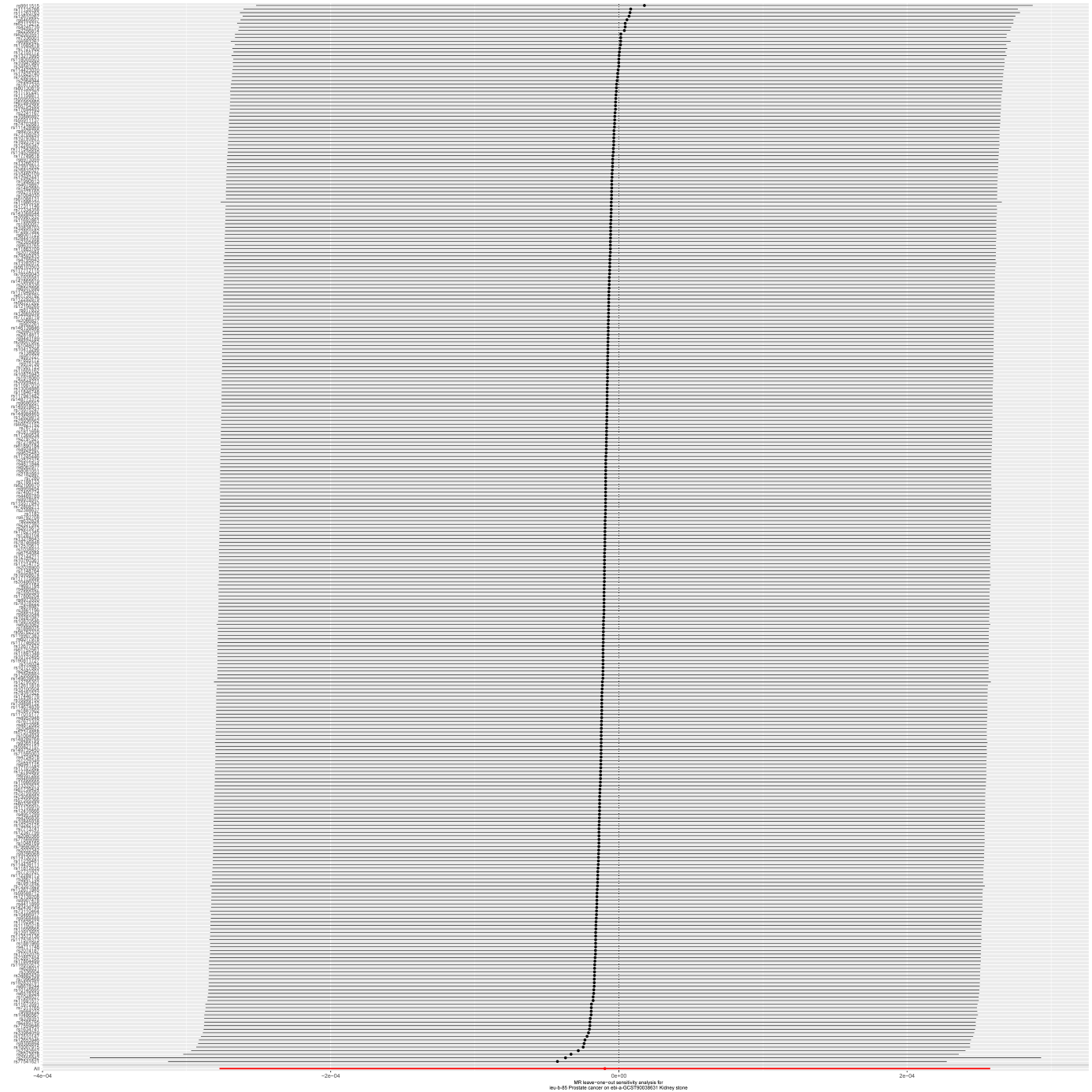


Caption: The x-axis represents the estimated causal effect after excluding each specific SNP, while the y-axis denotes the SNP identifier. The red horizontal line represents the estimated overall causal effect derived from all SNPs. The LOO results for all SNPs intersect the vertical line and align with the red horizontal line, indicating that no single SNP significantly disrupts the MR results.
